# *N*-(*p*-Tolyl­sulfon­yl)-l-asparagine

**DOI:** 10.1107/S1600536810004034

**Published:** 2010-02-06

**Authors:** Muhammad Nadeem Arshad, Hafiz Mubashar-ur-Rehman, Islam Ullah Khan, Muhammad Shafiq, Kong Mun Lo

**Affiliations:** a Materials Chemistry Laboratory, Department of Chemistry, GC University, Lahore 54000 Pakistan; b Department of Chemistry, University of Malaya, 50603 Kuala Lumpur, Malaysia

## Abstract

In the title compound, C_11_H_14_N_2_O_5_S, the amide O atom acts as a hydrogen-bond acceptor from a carboxyl­ate O atom and a secondary amino N atom. In addition, one of the sulfonyl O atoms and the carbonyl O atom of the carboxyl group also form hydrogen bonds with the primary amido N atom. These intermolecular hydrogen-bonding inter­actions give rise to a layer structure, with the layers parallel to the *ac* plane.

## Related literature

For the anti­bacterial and anti­cancer activity of l-asparagines, Wagastuma *et al.* (1983 ▶); Murphy & Stubbins (1980 ▶). For a related compound, see Arshad *et al.* (2009 ▶).
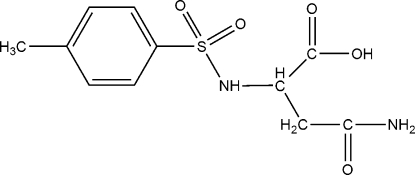


## Experimental

### 

#### Crystal data

C_11_H_14_N_2_O_5_S
*M* *_r_* = 286.30
Orthorhombic, 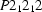

*a* = 8.7566 (6) Å
*b* = 22.900 (2) Å
*c* = 6.9692 (7) Å
*V* = 1397.5 (2) Å^3^
*Z* = 4
Mo *K*α radiation
μ = 0.25 mm^−1^
*T* = 296 K
0.37 × 0.11 × 0.07 mm

#### Data collection

Bruker APEXII CCD area-detector diffractometer
Absorption correction: multi-scan (*SADABS*; Sheldrick, 1996 ▶) *T*_min_ = 0.914, *T*_max_ = 0.983
8388 measured reflections
3206 independent reflections
2451 reflections with *I* > 2σ(*I*)
*R*_int_ = 0.029

#### Refinement

*R*[*F* ^2^ > 2σ(*F* ^2^)] = 0.042
*wR*(*F* ^2^) = 0.099
*S* = 1.02
3206 reflections
174 parameters
H-atom parameters constrained
Δρ_max_ = 0.18 e Å^−3^
Δρ_min_ = −0.27 e Å^−3^
Absolute structure: Flack (1983 ▶), 1338 Friedel pairs
Flack parameter: −0.03 (8)

### 

Data collection: *APEX2* (Bruker, 2008 ▶); cell refinement: *SAINT* (Bruker, 2008 ▶); data reduction: *SAINT*; program(s) used to solve structure: *SHELXS97* (Sheldrick, 2008 ▶); program(s) used to refine structure: *SHELXL97* (Sheldrick, 2008 ▶); molecular graphics: *X-SEED* (Barbour, 2001 ▶); software used to prepare material for publication: *publCIF* (Westrip, 2010 ▶).

## Supplementary Material

Crystal structure: contains datablocks I, global. DOI: 10.1107/S1600536810004034/bv2133sup1.cif

Structure factors: contains datablocks I. DOI: 10.1107/S1600536810004034/bv2133Isup2.hkl

Additional supplementary materials:  crystallographic information; 3D view; checkCIF report

## Figures and Tables

**Table 1:** Hydrogen-bond geometry (Å, °)

*D*—H⋯*A*	*D*—H	H⋯*A*	*D*⋯*A*	*D*—H⋯*A*
O4—H4⋯O5^i^	0.82	1.78	2.592 (2)	168
N1—H1⋯O5^ii^	0.86	2.06	2.852 (2)	154
N2—H2*A*⋯O3^iii^	0.86	2.12	2.924 (2)	155
N2—H2*B*⋯O2^ii^	0.86	2.06	2.901 (2)	166

Symmetry codes: (i) 
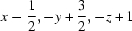
; (ii) 
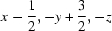
; (iii) 
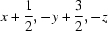
.

**Fig. 1.**
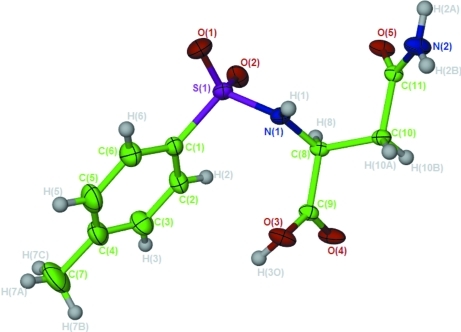
The molecular structure of N-(4-toluenesulfonyl)-L-asparagine showing 70% probability displacement ellipsoids and the atom numbering. Hydrogen atoms are drawn as spheres of arbitrary radius.

**Fig. 2.**
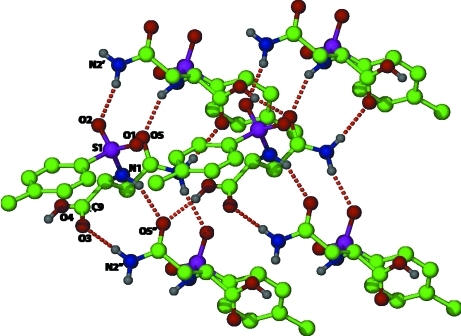
Crystal packing of the unit cell showing the hydrogen bonding interactions in the molecule.

## supplementary crystallographic information

### Comment

L-asparagine, amino acid derivatives of amino penicillin have been synthesized and reported as active antibacterial agents (Wagastuma *et al.*, 1983). In another study, different derivatives of asparagine have been synthesized and evaluated for their anticancer activities (Murphy & Stubbins *et al.*, 1980). Our group also involved in the synthesis of sulfonamide derivatives of different amino acids (Arshad *et al.*, 2009). In this sulfonamide derivative of L-asparagine (Fig. 1), the tetrahedral geometries at S1, C8 and C10 resulted in a twisted molecule in order to reduce steric hindrance. The molecules are linked together by hydrogen bonding between the amido oxygen O5 with the hydrogen atoms of the carboxylate oxygen O4 and the secondary amino nitrogen N1. In addition, one of the sulfonyl oxygen O2 and the carbonyl oxygen O3 also form hydrogen bonds with the primary amido nitrogen N2. These hydrogen bonding interactions produce a layer structure, with the layers propagated parallel to the *ac* plane (Fig. 2).

### Experimental

Asparagine (.25 g, 1.89 mmol) was dissolved in distilled water (10 ml) in a round bottom flask (25 ml). The pH of the solution was maintained at 8–9 using 1 *M* Na_2_CO_3_ solution. 4-Toluenesulfonyl chloride (0.361 g, 1.89 mmol) was suspended in the above solution and stirred at room temperature until all the 4-toluenesulfonyl chloride was consumed. The reaction was completed when the suspension turned to a clear solution. Upon completion of the reaction, the pH was adjusted 1–2, using 1 *M* HCl solution. The precipitate obtained was filtered, washed with distilled water, dried and recrystalized from methanol to yield white crystals.

### Refinement

Hydrogen atoms were placed at calculated positions (C–H 0.93 to 0.98 Å; O–H 0.82 Å, N–H 0.86 Å) and were treated as riding on their parent atoms, with *U*(H) set to 1.2–1.5 times *U*~eq~(C).

### Crystal data

**Table:**

C_11_H_14_N_2_O_5_S	*F*(000) = 600
*M**_r_* = 286.30	*D*_x_ = 1.361 Mg m^−^^3^
Orthorhombic, *P*2_1_2_1_2	Mo *K*α radiation, λ = 0.71073 Å
Hall symbol: P 2 2ab	Cell parameters from 2600 reflections
*a* = 8.7566 (6) Å	θ = 2.5–24°
*b* = 22.900 (2) Å	µ = 0.25 mm^−^^1^
*c* = 6.9692 (7) Å	*T* = 296 K
*V* = 1397.5 (2) Å^3^	Block, white
*Z* = 4	0.37 × 0.11 × 0.07 mm

### Data collection

**Table:**

Bruker APEXII CCD area-detector diffractometer	3206 independent reflections
Radiation source: fine-focus sealed tube	2451 reflections with *I* > 2σ(*I*)
graphite	*R*_int_ = 0.029
ω scans	θ_max_ = 27.5°, θ_min_ = 2.5°
Absorption correction: multi-scan (*SADABS*; Sheldrick, 1996)	*h* = −11→11
*T*_min_ = 0.914, *T*_max_ = 0.983	*k* = −26→29
8388 measured reflections	*l* = −9→9

### Refinement

**Table:**

Refinement on *F*^2^	Secondary atom site location: difference Fourier map
Least-squares matrix: full	Hydrogen site location: inferred from neighbouring sites
*R*[*F*^2^ > 2σ(*F*^2^)] = 0.042	H-atom parameters constrained
*wR*(*F*^2^) = 0.099	*w* = 1/[σ^2^(*F*_o_^2^) + (0.0521*P*)^2^ + ] where *P* = (*F*_o_^2^ + 2*F*_c_^2^)/3
*S* = 1.02	(Δ/σ)_max_ < 0.001
3206 reflections	Δρ_max_ = 0.18 e Å^−^^3^
174 parameters	Δρ_min_ = −0.27 e Å^−^^3^
0 restraints	Absolute structure: Flack (1983), 1328 Friedel pairs
Primary atom site location: structure-invariant direct methods	Flack parameter: −0.03 (8)

### Special details

**Table:**

Geometry. All e.s.d.'s (except the e.s.d. in the dihedral angle between two l.s. planes) are estimated using the full covariance matrix. The cell e.s.d.'s are taken into account individually in the estimation of e.s.d.'s in distances, angles and torsion angles; correlations between e.s.d.'s in cell parameters are only used when they are defined by crystal symmetry. An approximate (isotropic) treatment of cell e.s.d.'s is used for estimating e.s.d.'s involving l.s. planes.
Refinement. Refinement of *F*^2^ against ALL reflections. The weighted *R*-factor *wR* and goodness of fit *S* are based on *F*^2^, conventional *R*-factors *R* are based on *F*, with *F* set to zero for negative *F*^2^. The threshold expression of *F*^2^ > σ(*F*^2^) is used only for calculating *R*-factors(gt) *etc*. and is not relevant to the choice of reflections for refinement. *R*-factors based on *F*^2^ are statistically about twice as large as those based on *F*, and *R*- factors based on ALL data will be even larger.

### Fractional atomic coordinates and isotropic or equivalent isotropic displacement parameters (Å^2^)

**Table:**

	*x*	*y*	*z*	*U*_iso_*/*U*_eq_	
S1	0.95716 (6)	0.62903 (3)	0.10823 (10)	0.04939 (18)	
O1	0.9573 (2)	0.61117 (9)	−0.0862 (3)	0.0778 (6)	
O2	1.09832 (17)	0.64258 (8)	0.2020 (3)	0.0636 (5)	
O3	0.62170 (16)	0.68014 (8)	0.3996 (2)	0.0554 (4)	
O4	0.76473 (19)	0.73119 (9)	0.6017 (2)	0.0629 (5)	
H4	0.7019	0.7200	0.6808	0.094*	
O5	1.08999 (14)	0.79700 (8)	0.1128 (2)	0.0493 (4)	
N1	0.85411 (17)	0.68699 (8)	0.1208 (3)	0.0392 (4)	
H1	0.7966	0.6960	0.0253	0.047*	
N2	0.90525 (19)	0.82712 (9)	−0.0810 (3)	0.0522 (5)	
H2A	0.9703	0.8367	−0.1680	0.063*	
H2B	0.8093	0.8323	−0.1008	0.063*	
C1	0.8685 (3)	0.57415 (11)	0.2442 (4)	0.0490 (6)	
C2	0.8893 (3)	0.57211 (12)	0.4400 (4)	0.0619 (7)	
H2	0.9511	0.5994	0.5013	0.074*	
C3	0.8171 (4)	0.52899 (16)	0.5433 (5)	0.0807 (10)	
H3	0.8303	0.5277	0.6757	0.097*	
C4	0.7259 (4)	0.48771 (14)	0.4570 (6)	0.0796 (10)	
C5	0.7061 (4)	0.49096 (16)	0.2613 (7)	0.0896 (11)	
H5	0.6442	0.4637	0.2002	0.107*	
C6	0.7762 (3)	0.53379 (13)	0.1549 (5)	0.0712 (9)	
H6	0.7613	0.5355	0.0229	0.085*	
C7	0.6559 (5)	0.43887 (16)	0.5732 (7)	0.1317 (18)	
H7A	0.5955	0.4145	0.4908	0.198*	
H7B	0.5921	0.4551	0.6719	0.198*	
H7C	0.7355	0.4160	0.6309	0.198*	
C8	0.8551 (2)	0.72435 (9)	0.2877 (3)	0.0357 (5)	
H8	0.9551	0.7208	0.3497	0.043*	
C9	0.7331 (2)	0.70850 (10)	0.4343 (3)	0.0375 (5)	
C10	0.8332 (2)	0.78799 (10)	0.2275 (3)	0.0416 (5)	
H10A	0.7319	0.7931	0.1735	0.050*	
H10B	0.8420	0.8131	0.3389	0.050*	
C11	0.9521 (2)	0.80490 (9)	0.0805 (3)	0.0362 (5)	

### Atomic displacement parameters (Å^2^)

**Table:**

	*U*^11^	*U*^22^	*U*^33^	*U*^12^	*U*^13^	*U*^23^
S1	0.0400 (3)	0.0489 (3)	0.0592 (4)	0.0011 (3)	0.0150 (3)	−0.0021 (3)
O1	0.0992 (14)	0.0717 (13)	0.0624 (12)	0.0039 (11)	0.0320 (12)	−0.0157 (11)
O2	0.0324 (8)	0.0578 (11)	0.1005 (15)	0.0023 (7)	0.0067 (8)	0.0111 (11)
O3	0.0385 (8)	0.0806 (12)	0.0471 (9)	−0.0149 (8)	0.0005 (7)	0.0108 (10)
O4	0.0571 (10)	0.0946 (15)	0.0369 (9)	−0.0274 (9)	0.0162 (8)	−0.0073 (10)
O5	0.0301 (7)	0.0789 (12)	0.0388 (8)	−0.0034 (7)	−0.0020 (7)	0.0164 (10)
N1	0.0368 (8)	0.0467 (11)	0.0342 (9)	0.0025 (8)	0.0010 (8)	0.0005 (10)
N2	0.0325 (8)	0.0808 (15)	0.0432 (11)	0.0045 (9)	0.0011 (8)	0.0199 (11)
C1	0.0413 (11)	0.0409 (14)	0.0646 (16)	0.0019 (10)	0.0084 (11)	−0.0013 (13)
C2	0.0677 (16)	0.0459 (16)	0.072 (2)	−0.0084 (13)	−0.0039 (13)	0.0062 (14)
C3	0.096 (2)	0.067 (2)	0.079 (2)	−0.0022 (19)	0.0075 (17)	0.0203 (19)
C4	0.074 (2)	0.046 (2)	0.119 (3)	−0.0002 (16)	0.0206 (19)	0.017 (2)
C5	0.076 (2)	0.059 (2)	0.134 (3)	−0.0230 (16)	0.006 (2)	−0.010 (2)
C6	0.0749 (18)	0.0609 (19)	0.078 (2)	−0.0156 (16)	0.0060 (15)	−0.0088 (17)
C7	0.127 (3)	0.074 (3)	0.195 (5)	−0.013 (2)	0.033 (4)	0.059 (3)
C8	0.0277 (9)	0.0456 (13)	0.0338 (11)	−0.0016 (9)	0.0021 (8)	0.0037 (10)
C9	0.0315 (9)	0.0479 (14)	0.0332 (11)	0.0011 (9)	0.0000 (8)	0.0078 (10)
C10	0.0390 (11)	0.0457 (14)	0.0400 (12)	0.0033 (10)	0.0086 (9)	0.0032 (11)
C11	0.0339 (9)	0.0393 (11)	0.0353 (11)	−0.0010 (9)	0.0012 (9)	0.0021 (10)

### Geometric parameters (Å, °)

**Table:**

S1—O1	1.416 (2)	C3—C4	1.376 (5)
S1—O2	1.4322 (18)	C3—H3	0.9300
S1—N1	1.6074 (18)	C4—C5	1.377 (5)
S1—C1	1.755 (3)	C4—C7	1.511 (4)
O3—C9	1.197 (2)	C5—C6	1.374 (5)
O4—C9	1.307 (2)	C5—H5	0.9300
O4—H4	0.8200	C6—H6	0.9300
O5—C11	1.242 (2)	C7—H7A	0.9600
N1—C8	1.444 (3)	C7—H7B	0.9600
N1—H1	0.8600	C7—H7C	0.9600
N2—C11	1.302 (3)	C8—C9	1.522 (3)
N2—H2A	0.8600	C8—C10	1.529 (3)
N2—H2B	0.8600	C8—H8	0.9800
C1—C6	1.376 (4)	C10—C11	1.511 (3)
C1—C2	1.378 (4)	C10—H10A	0.9700
C2—C3	1.376 (4)	C10—H10B	0.9700
C2—H2	0.9300		
			
O1—S1—O2	119.92 (11)	C5—C6—C1	119.8 (3)
O1—S1—N1	106.93 (11)	C5—C6—H6	120.1
O2—S1—N1	106.30 (10)	C1—C6—H6	120.1
O1—S1—C1	108.07 (13)	C4—C7—H7A	109.5
O2—S1—C1	106.91 (12)	C4—C7—H7B	109.5
N1—S1—C1	108.27 (10)	H7A—C7—H7B	109.5
C9—O4—H4	109.5	C4—C7—H7C	109.5
C8—N1—S1	122.00 (14)	H7A—C7—H7C	109.5
C8—N1—H1	119.0	H7B—C7—H7C	109.5
S1—N1—H1	119.0	N1—C8—C9	113.27 (17)
C11—N2—H2A	120.0	N1—C8—C10	110.06 (17)
C11—N2—H2B	120.0	C9—C8—C10	108.87 (17)
H2A—N2—H2B	120.0	N1—C8—H8	108.2
C6—C1—C2	120.2 (3)	C9—C8—H8	108.2
C6—C1—S1	119.8 (2)	C10—C8—H8	108.2
C2—C1—S1	120.0 (2)	O3—C9—O4	124.65 (19)
C3—C2—C1	118.8 (3)	O3—C9—C8	124.49 (19)
C3—C2—H2	120.6	O4—C9—C8	110.84 (17)
C1—C2—H2	120.6	C11—C10—C8	110.12 (17)
C4—C3—C2	122.1 (3)	C11—C10—H10A	109.6
C4—C3—H3	119.0	C8—C10—H10A	109.6
C2—C3—H3	119.0	C11—C10—H10B	109.6
C3—C4—C5	118.0 (3)	C8—C10—H10B	109.6
C3—C4—C7	120.7 (4)	H10A—C10—H10B	108.2
C5—C4—C7	121.3 (4)	O5—C11—N2	121.34 (19)
C6—C5—C4	121.1 (3)	O5—C11—C10	120.65 (19)
C6—C5—H5	119.4	N2—C11—C10	118.00 (17)
C4—C5—H5	119.4		
			
O1—S1—N1—C8	−166.24 (16)	C7—C4—C5—C6	−176.8 (3)
O2—S1—N1—C8	−37.03 (17)	C4—C5—C6—C1	0.3 (5)
C1—S1—N1—C8	77.52 (17)	C2—C1—C6—C5	−0.7 (4)
O1—S1—C1—C6	−19.0 (3)	S1—C1—C6—C5	−179.7 (3)
O2—S1—C1—C6	−149.3 (2)	S1—N1—C8—C9	−91.79 (18)
N1—S1—C1—C6	96.5 (2)	S1—N1—C8—C10	146.07 (15)
O1—S1—C1—C2	162.1 (2)	N1—C8—C9—O3	−19.7 (3)
O2—S1—C1—C2	31.7 (3)	C10—C8—C9—O3	103.1 (2)
N1—S1—C1—C2	−82.4 (2)	N1—C8—C9—O4	161.76 (19)
C6—C1—C2—C3	0.3 (4)	C10—C8—C9—O4	−75.4 (2)
S1—C1—C2—C3	179.3 (2)	N1—C8—C10—C11	−54.8 (2)
C1—C2—C3—C4	0.5 (5)	C9—C8—C10—C11	−179.54 (17)
C2—C3—C4—C5	−1.0 (5)	C8—C10—C11—O5	−53.0 (3)
C2—C3—C4—C7	176.4 (3)	C8—C10—C11—N2	126.1 (2)
C3—C4—C5—C6	0.6 (6)		

### Hydrogen-bond geometry (Å, °)

**Table:**

*D*—H···*A*	*D*—H	H···*A*	*D*···*A*	*D*—H···*A*
O4—H4···O5^i^	0.82	1.78	2.592 (2)	168
N1—H1···O5^ii^	0.86	2.06	2.852 (2)	154
N2—H2A···O3^iii^	0.86	2.12	2.924 (2)	155
N2—H2B···O2^ii^	0.86	2.06	2.901 (2)	166

Symmetry codes: (i) *x*−1/2, −*y*+3/2, −*z*+1; (ii) *x*−1/2, −*y*+3/2, −*z*; (iii) *x*+1/2, −*y*+3/2, −*z*.
